# Drug related problems in older adults living with dementia

**DOI:** 10.1371/journal.pone.0236830

**Published:** 2020-07-31

**Authors:** Sirasa Ruangritchankul, Nancye M. Peel, Leila Shafiee Hanjani, Leonard C. Gray

**Affiliations:** 1 Centre for Health Services Research, Faculty of Medicine, The University of Queensland, Brisbane, QLD, Australia; 2 Division of Geriatric Medicine, Department of Medicine, Faculty of Medicine, Ramathibodi Hospital, Mahidol University, Bangkok, Thailand; Nord University, NORWAY

## Abstract

**Background:**

Compared with those without dementia, older patients with dementia admitted to acute care settings are at higher risk for triad combination of polypharmacy (PP), potentially inappropriate medication (PIM), and drug-drug interaction (DDI), which may consequently result in detrimental health. The aims of this research were to assess risk factors associated with triad combination of PP, PIM and DDI among hospitalized older patients with dementia, and to assess prevalence and characteristics of PP, PIM and DDI in this population.

**Methods:**

In this retrospective cross-sectional study, 416 older inpatients diagnosed with dementia and referred for specialist geriatric consultation at a tertiary hospital in Brisbane, Australia during 2006–2016 were enrolled. Patients were categorized into two groups according to their exposure to the combination of PP, PIM and DDI: ‘triad combination’ and ‘non-triad combination’. Data were collected using the interRAI Acute Care (AC) assessment instrument. Independent risk factors of exposure to the triad combination were evaluated using bivariate and multivariate logistic regression analyses.

**Results:**

Overall, 181 (43.5%) were classified as triad combination group. The majority of the population took at least 1 PIM (56%) or experienced at least one potential DDI (76%). Over 75% of the participants were exposed to polypharmacy. The most common prescribed PIMs were antipsychotics, followed by benzodiazepines. The independent risk factors of the triad combination were the presence of atrial fibrillation diagnosis and higher medications use in cardiac therapy, psycholeptics and psychoanaleptics.

**Conclusions:**

The exposure to triad combination of PP, PIM and DDI are common among people with dementia as a result of their vulnerable conditions and the greater risks of adverse events from medications use. This study identified the use of cardiac therapy, psycholeptics and psychoanaleptics as predictors of exposure to PP, PIM and DDI. Therefore, use of these medications should be carefully considered and closely monitored. Furthermore, comprehensive medication reviews to optimize medication prescribing should be initiated and continually implemented for this vulnerable population.

## Introduction

During the recent decades, the increasing aging population and the great gains in life expectancy have resulted in raised prevalence of neurodegenerative diseases including dementia. Globally, the number of people affected by dementia was almost 50 million in 2017 and is projected to increase to 131.5 million by 2050 [1]. In Australia, the number of people living with dementia was 447,115 in 2019. In the next four decades, the number will over double to reach an estimated number of 1,076,129 by 2058 [2].

Dementia is a term used to describe clinical syndromes associated with irreversibly progressive deterioration of multiple higher cortical functions, including language, memory, calculation, comprehension, thinking, learning skills and appropriate judgment, leading to interfering with functional ability and daily life [3,4]. Due to the deterioration of cortical functions, especially in memory, people with dementia may take the medicine repeatedly or wrong medications, resulting in drug related problems (DRPs) and increased medication use. These problems have devastating effects on health outcomes, such as adverse drug events, morbidity and mortality. Dementia is the greatest risk factor of disability and premature mortality in Australian older people aged 65 years and older [5,6]. In 2017, dementia was the second-leading cause of death in Australia, just after coronary artery diseases [7]. The average survival time of people living with dementia has ranged from 3 to 13 years [8,9]. Finally, the economic impact of dementia has markedly been concerning. The estimated cost of dementia in Australia was over $15 billion in 2019 and is estimated to increase to over $36.8 billion by 2056 [6].

Dementia is usually associated with the development of three or more chronic diseases including hypertension, stroke, diabetes and coronary heart disease, resulting in increased health care needs and medication use [10–16]. Dementia and demented-related complications are common causes for unplanned visits to acute care sectors and then contribute to adverse consequences including falls, superimposed delirium, frailty, malnutrition, increased mortality rate and longer hospital stay [17–26]. In Australia, approximately 83,000 people with dementia are admitted to acute care settings each year [27]. In previous studies, between 15% to 40% of admitted patients were diagnosed with dementia, and they accounted for 10% of acute care bed days [28–32]. With regard to medication use, people with dementia are more likely to take many medications for treatment of cognitive problems, psycho-behavioral symptoms and multiple comorbidities, compared with people without dementia [33,34]. Most geriatric patients with dementia usually consume a mean of five or more medications, commonly defined as polypharmacy (PP) [35–39]. Although taking multiple medications may be necessary for the management of dementia and chronic diseases, the use of multiple medications can lead to greater risk of DRPs, including potentially inappropriate medications (PIMs), drug-drug interactions (DDIs), and adverse drug reactions (ADRs) [40–45]. Apart from polypharmacy, age-related pharmacokinetic and pharmacodynamic changes can pose higher risk for undesirable DRPs [46,47]. Furthermore, older people with dementia have an alteration of blood-brain permeability and levels of endogenous neurotransmitters which may increase their sensitivity to neurological effects of medications, leading to greater experience of ADRs [46,48,49]. The PP and DRPs result in an increased risk of hospital admission, morbidity, mortality and health care burdens [50–53]. These problems tend to occur more frequently and are more serious in geriatric population with dementia due to their vulnerability than in younger population. The exposure to the combination of PP and DRPs is commonly identified in older patients with dementia. Many previous studies have reported high prevalence of PP and DRPs among older patients with dementia admitted to hospitals. Studies have reported the exposure of 82–98% of this vulnerable population to the PP [54–56], 22.4–96% to the PIM [56–59] and 43.2% to the DDI [60].

However, although many studies have assessed aspects related to PP, PIM or DDI, no previous study has explored prevalence and associated risk factors for the exposure to three criteria or triad combination of PP, PIM and DDI among the older people with dementia admitted in acute care setting.

The first aim of the current study was to evaluate risk factors associated with the exposure to triad combination of PP, PIM and DDI among patients with dementia. The other aim was to assess prevalence and characteristics of PP, PIM and DDI and to determine the most frequently implicated medications in the context of this vulnerable population.

## Materials and methods

### Study design, setting and participants

The present study was a secondary analysis of data from the Comprehensive electronic Geriatric Assessment database [61] of patients referred for specialist geriatric consultation at Princess Alexandra Hospital, Brisbane, Australia during the period from January 1, 2006 to December 31, 2016. The interRAI Acute Care (AC) tool was used for comprehensive geriatric assessment [62]. The criteria for selection of individuals into the study were patients aged 65 years and over with dementia based on the medical records of disease diagnosis “dementia” combined with cognitive performance scale (CPS) scores ranging from 2 to 6, derived from the interRAI Acute Care assessment. The participants were categorized into two groups: triad combination group and non-triad combination group. The triad combination group comprised those patients exposed to all three criteria of PP, PIM and DDI. The non-triad combination group comprised those without exposure to the combination of three criteria of PP, PIM and DDI.

### Data collection and measurement tools

In this study, all data were de-identified health data provided from the interRAI Acute Care (AC) database [62,63]. A standardized interRAI Acute Care (AC) assessment tool was used to support comprehensive geriatric assessment (CGA) in the acute setting for the older patients [61] at admission, trained nurse assessors gathered comprehensive health information data including physical, physiological-behavioral and socio-economic status, and conducted many clinical assessments for example, cognitive status, psycho-behavioural conditions and activities of daily living. If patients were not able to provide the medical information, a relative or a proxy informant could offer the required information. The individual variables (covariates) included baseline characteristics, living situation, primary diagnosis, comorbidities, geriatric syndrome, activity of daily living (ADL), instrumental activity of daily living (IADL), moods and behavioral patterns, cognitive functions, service utilization and all prescribed medications [62]. Within 24 hours of admission, trained nurse assessors gathered and recorded all health data and lists of prescribed medications. The participant’ s cognitive function was evaluated by embedded computer algorithms of InterRAI Acute Care (AC), which interpreted collected clinical data such as moods, behavior, and cognitive functions to create a CPS, correlated with Mini-Mental State Examination (MMSE), with scores ranging from 0 (intact cognition) to 6 (very severe cognitive impairment) [64]. Functional capacity and levels of dependence were evaluated by short ADL scale at admission and premorbid IADLs scale which were also embedded in InterRAI Acute Care (AC) database [63,65]. The ADL short-form scale at admission evaluates obtained assistance in performing four activities including eating, personal hygiene, toilet use and walking, ranging from 0 to 16 with higher scores indicating greater level of incapacity [63,66]. Premorbid IADL scale summarized the performance on seven IADL items (housework, meal preparation, shopping, finances, transport, phone use and medication management). Performance scores for each item range from 0 (independent) to 6 (total dependence). The summed scores were analysed to generate a scale ranging from 0 to 42, with higher scores indicating a greater level of dependence [63,66]. With regard to medication data, the medication name, frequency, route of administration and doses were recorded in the databases. All recorded medications were classified by Anatomical Therapeutic Chemical (ATC) codes [67], following recommendation by the World Health Organization, as the international standard to present medication usage data. Finally, the prevalence and characteristics of PP, PIM and DDI were determined by a geriatrician and/or pharmacist using following definitions.

### Definitions

#### Drug-Related Problem (DRP)

DRP is defined as any undesirable circumstance involving medication therapy which potentially interferes with desired health consequences [68]. In the current study, DRPs consist of presence of PIM and DDI.

#### Polypharmacy (PP)

PP is defined as the simultaneous consumption of five or more prescribed medications, based upon the number of regular prescribed medications documented in the InterRAI Acute Care (AC) database [69].

#### Potentially Inappropriate Medication (PIM)

By definition, PIM is a medication that should be avoided in an individual due to the health risks outweighing the clinical benefits [70]. In this study, PIMs were determined using the 2019 American Geriatrics Society (AGS) Beers Criteria^®^ (AGS Beers Criteria^®^), which are the fifth updated of the AGS Beers Criteria^®^ [71]. In the present study, PIMs independent of medical condition and PIMs that should be avoided in the older adults due to drug-disease or drug-syndrome interactions were determined.

#### Drug-Drug Interaction (DDI)

The definition of DDI is a situation in which one substance affects the activity of another drug when both are used simultaneously, regardless whether adverse events will occur or not [72]. All prescribed medications were analyzed by Micromedex Drug Interaction Database [73] to determine drug interactions which were considered only single pairwise of drug combination. In this study, potential drug interactions were reported in terms of severity and interaction effect. With respect to severity rating risk, DDIs were categorized into four classifications: minor, moderate, major and contraindicated DDIs [73]. Only the risk of potential adverse outcomes related to drug interactions were explored, but clinical relevant outcomes were not included.

### Statistical analysis

Statistical analyses were performed using the SPSS for Windows Software Package, Version 25 (SPSS Inc., Chicago, Ill., USA). To describe population characteristics and medication profiles, the percentage was used for categorical variables and mean ± standard deviation (SD) or median ± interquartile range (IQR) was used for continuous variables. Pearson’s chi-squared test or Fisher’s exact for categorical variables and Unpaired Student’s t-test or Mann-Whitney U test for continuous variables were performed to compare baseline characteristics, physical and cognitive function, medication profiles between triad combination group and non-triad combination group. Univariate logistic regression analysis was used first to evaluate the effect of variables on the exposure to triad combination of PP, PIM and DDI. Factors determined as significant variables from univariate logistic analysis (significance set at p<0.05 level), age, gender, number of comorbidity, short ADL scale at admission and premorbid IADLs were included in the final multivariate logistic regression analysis to estimate independent risk factors associated with the exposure to triad combination of PP, PIM and DDI. Additionally, subgroup analyses were performed to evaluate risk factors associated with greater exposure to each criterion of PP, PIM and DDI using bivariate and multivariate logistic regression analyses. The results from logistic regression analyses were presented as odd ratios (ORs) and 95% confidence intervals (CIs). Statistical significance levels for variables were determined at the level of p<0.05.

The present study was a secondary retrospective cross-sectional analysis of de-identified data provided by the interRAI Acute Care (AC) assessment database. The researchers did not require to identify the data and to contact the participants. Exemption from ethics approval was obtained from the Metro South Human Research Ethics Committee and Human Research.

## Results

### Baseline and clinical characteristics

Of 2,992 older patients age 65 years and older referred for geriatric consultation services at Princess Alexandra Hospital from 2006 to 2016, a total of 416 older patients with dementia (13.9%) were enrolled. The eligible participants were categorized into two groups: triad combination group and non-triad combination group. Overall, 181 (43.5%) participants and 235 (56.5%) participants were classified as triad combination group and non-triad combination group respectively. The descriptive analysis and a comparison of population study in terms of baseline and clinical characteristics between the two groups are presented in Table 1.

**Table 1:** Demographic and clinical characteristics of the population.

Characteristics	Triad combination (n = 181) N (%)	Non-triad combination (n = 235) N (%)	*P* value
Age in years, mean (SD)	82.5 (7.0)	82.7 (7.2)	0.791^#^
65–75 years	33 (18.2)	44 (18.7)	
> 75 years	148 (81.8)	191 (81.3)	
**Sex**			
Male	89 (49.2)	103 (43.8)	0.279*
Female	92 (50.8)	132 (56.2)	
**Marital status**			
Single	8 (4.4)	12 (5.1)	0.289*
Married	76 (42.0)	78 (33.2)	
Widowed	73 (40.3)	114 (48.5)	
Divorced or separated	24 (13.3)	31 (13.2)	
Living alone	55 (30.4)	95 (40.4)	0.035*
**Admission sources**			
Community	152 (84.0)	213 (90.6)	0.059*
Hospital	4 (2.2)	6 (2.6)	
RACF	25 (13.8)	16 (6.8)	
Length of hospital stay in days, median (IQR)	32 (22, 50.5)	31 (21, 44)	0.408^+^
**Discharge destination**			
Community	75 (41.4)	92 (39.1)	0.640*
Hospital	23 (12.7)	24 (10.2)	
RACF	74 (40.9)	102 (43.4)	
Death	9 (5.0)	17 (7.2)	
**Comorbidities**			
No. of comorbidities/person, mean (SD)	5.8 (1.9)	5.2 (2.0)	0.001^#^
No. of comorbidities > 3/ person	156 (86.2)	187 (79.6)	0.079*
Hypertension	93 (51.4)	120 (51.1)	0.949*
Diabetes mellitus	46 (25.4)	36 (15.3)	0.010*
Coronary artery disease	65 (35.9)	62 (26.4)	0.036*
Atrial fibrillation	47 (26.0)	34 (14.5)	0.003*
Ischemic stroke	37 (20.4)	39 (16.6)	0.314*
Dyslipidemia	50 (27.6)	53 (22.6)	0.235*
Malignancy	39 (21.5)	48 (20.4)	0.780*
Infection	71 (39.2)	86 (36.6)	0.583*
GERD	57 (31.5)	56 (23.8)	0.082*
Osteoporosis	36 (19.9)	29 (12.3)	0.036*
Delirium	35 (10.5)	17 (7.2)	0.241*
**Primary diagnosis**			
Dementia with psycho-behavioral symptoms	54 (29.8)	70 (29.8)	1.000*
Fall	44 (24.3)	51 (21.7)	0.560*
Urinary tract infection	32 (17.7)	32 (13.6)	0.476*
Weight (kg), median (IQR)	60 (52, 73)	60 (52, 70)	0.884^+^
BMI (kg/m^2^), mean (SD)	23.4 (4.5)	22.8 (4.5)	0.167^#^
**Geriatric syndrome**			
Cognitive status at admission^a^			
Mild-moderate impairment (2–4)	141 (77.9)	187 (79.6)	0.698*
Severe impairment (5–6)	40 (22.1)	48 (20.4)	
Short ADL scale at admission, median (IQR)^b^	6 (3.5, 11)	7 (2, 10)	0.130^+^
Premorbid IADLs, mean (SD)^c^	31.8 (11.2)	29.3 (12.8)	0.032^#^
Weight loss^d^	37 (20.4)	56 (23.8)	0.411*
Urinary incontinence	122 (67.4)	150 (63.8)	0.448*
Visual problems	84 (46.4)	89 (37.9)	0.080*
Auditory problems	94 (51.9)	111 (47.2)	0.342*

**Data are presented as** mean (standard deviation), n (%), or median (interquartile range).

* Chi-square test

^#^ Student’s t-test

^+^ Mann–Whitney U test.

**Abbreviations:** RACF, residential aged care facilities; SD, standard deviation; IQR, interquartile range; GERD, gastroesophageal reflux disease; kg, kilogram; m, meter; BMI, body mass index; ADL, activities of daily living; IADL, instrumental ADL.

^a^ Based on the CPS, which ranges from 0 (intact cognition) to 6 (very severe cognitive impairment); [64] cognitively impaired patients were defined as CPS scores ≥ 2, corresponding to a mean Mini Mental State Examination score of < 24 [64]

^b^The ADL short-form scale comprises four items (personal hygiene, walking, toilet use and eating); range is 0–16, with higher scores reflecting greater level of dependency [63,66].

^c^ Premorbid IADL scale summarizes the performance on seven IADL items (meal preparation, housework, finances, medication management, phone use, shopping, and transport). The scale has a range from 0 to 42, with higher scores indicating greater dependence [63,66].

^d^ Loss of ≥ 5% bodyweight in the 30 days before admission or ≥ 10% in the 180 days before admission.

The majority of the participants were female (n = 224; 53.8%). The age ranged from 65 to 101 years with mean of 82.3 years (SD 7.1 years), which was not significantly different between the two groups. Over half of the participants were widowed, divorced or separated. The majority of the total population (> 80%) resided in their own homes, but less than half of them lived alone. In the current study, it was found that the number of the patients lived alone in triad combination group was significantly lower as compared to the non-triad combination group. Approximately one tenth were admitted from residential aged care facilities. The median length of hospital admission was not significantly different between the two groups (p = 0.408). With regard to discharge destination, over 40% were discharged to RACF. In both groups, approximately 6% ended their lives in hospital. Dementia with psycho-behavioural symptoms (29.8%), falls (22.8%) and urinary tract infection (15.4%) were the main causes of the current hospitalization. The participants in the triad combination group had substantially more comorbidities as compared to the non-triad combination group (p = 0.001). More than three quarters of the total population had three or more comorbidities. In both groups, the participants were more likely to have hypertension, infection and coronary artery disease. It was found that the number of participants having diabetes, coronary artery disease, atrial fibrillation and osteoporosis in the triad combination were markedly higher comparing with the non-triad combination group (p<0.05). With respect to functional capacity and geriatric syndrome, the majority of the participants were more likely to have impaired hearing (49.3%), urinary incontinence (65.4%), and mild to moderate cognitive impairment (78.8%) with CPS scores ranging from 2 to 4. The participants in the triad combination group had a tendency to be of greater dependence in IADL as compared to those in the non-triad combination group [31.8 (SD 11.2) in the triad combination group vs 29.3 (SD 12.8) in the non-triad combination group, p = 0.032].

### Medication use

The most common prescribed medications categorized by ATC codes are presented in Table 2. The total number of regular prescribed medications was 3,027. The number of regular prescribed medications per person ranged from 0 to 19. Two participants in the non-triad combination group and six in the triad combination group consumed more than 15 medications. The median number of prescribed medications was 8 (IQR 6–10 medications) in the triad combination group and 6 (IQR 4–8 medications) in the non-triad combination group. This was significantly different between the two groups (p< 0.001). Three most frequently prescribed medication categories were antithrombotic agents (76.4%), followed by drugs for constipation (41.8%) and agent acting on the renin-angiotensin system (39.4%). In the triad combination group, prescriptions of cardiac therapy, diuretics, psycholeptics and psychoanaleptics were substantially higher as compared to the non-triad combination group (p<0.001). Up to 10% of the total participants were using anti-dementia medications which consisted of three cholinesterase inhibitors and one N-methyl-D-aspartate (NMDA) receptor antagonists.

**Table 2:** Prescribed medications use of the population according to ATC system.

Characteristics	Triad combination (n = 181) N (%)	Non-triad combination (n = 235) N (%)	*P* value
Total number of medications	1577 (100)	1450 (100)	
No. of prescribed medications/person, median (IQR)	8 (6, 10)	6 (4, 8)	<0.001^+^
**Prescribed medications according to ATC classes and codes**			
A02 Drugs for acid related disorders	70 (38.7)	65 (27.7)	0.017*
A06 Drugs for constipation	77 (42.5)	97 (41.3)	0.795*
A10 Drug used in diabetes	34 (18.8)	24 (10.2)	0.012*
A11 Vitamins	69 (38.1)	87 (37.0)	0.818*
A12 Mineral supplements	75 (41.4)	68 (28.9)	0.008*
B01 Antithrombotic agents	144 (79.6)	174 (74.0)	0.189*
C01 Cardiac therapy	51 (28.2)	21 (8.9)	< 0.001*
C02 Antihypertensives	11 (6.1)	5 (2.1)	0.038*
C03 Diuretics	54 (29.8)	35 (14.9)	<0.001*
C07 Beta blocking agents	66 (36.5)	62 (26.4)	0.027*
C08 Calcium channel blockers	32 (17.7)	42 (17.9)	0.959*
C09 Agents acting on the renin- angiotensin system	84 (46.4)	80 (34.0)	0.011*
C10 Lipid modifying agents	76 (42.0)	70 (29.8)	0.010*
D Dermatologicals	6 (3.3)	13 (9.3)	0.035*
H02 Corticosteroids	15 (8.3)	18 (7.7)	0.060*
**Prescribed medications according to ATC classes and codes**			
H03 Thyroid therapy	29 (16.0)	21 (8.9)	0.028*
J01 Antibacterial drugs	44 (24.3)	44 (18.7)	0.167*
N02 Analgesics	77 (42.5)	80 (34.0)	0.076*
N03 Antiepileptics	19 (10.5)	10 (4.3)	0.013*
N05 Psycholeptics	97 (53.6)	45 (19.1)	< 0.001*
N05A Antipsychotics	82 (45.3)	37 (15.7)	< 0.001*
N05B-N05C Anxiolytics, sedatives and hypnotics	31 (17.1)	12 (5.1)	< 0.001*
N06 Psychoanaleptics	74 (40.9)	49 (20.9)	< 0.001*
N06A Antidepressants	65 (35.9)	37 (15.7)	< 0.001*
N06D Anti-dementia drugs	20 (11.0)	16 (6.8)	0.127*
R03 Drugs for obstructive airway diseases	19 (10.5)	22 (9.4)	0.700*

**Data are presented as** n (%), or median (interquartile range).

* Chi-square test

^+^ Mann–Whitney U test.

**Abbreviations:** IQR, interquartile range; ATC, anatomical therapeutic chemical.

### PP, PIM and DDI

In both groups, approximately 78% of the participants were exposed to PP. Approximately 76% of the participants experienced at least 1 potential DDI, and 233 participants (56%) had at least 1 PIM, as shown in Table 3.

**Table 3:** Prevalence of medication issues in the study population.

Characteristics	N (%)
**Number of participants with the different combinations of criteria of PP, PIM, DDI**	416 (100)
None	43 (10.3)
Only PP	20 (4.8)
Only PIM	23 (5.5)
Only DDI	7 (1.7)
PP + PIM	14 (3.4)
PP + DDI	112 (26.9)
PIM + DDI	16 (3.8)
PP + PIM + DDI	181 (43.5)
**Potentially inappropriate medications (PIMs), elderly person**	416 (100)
0	183 (44.0)
1	141 (33.9)
2	65 (15.6)
3	23 (5.5)
4	4 (1.0)
**Drug-drug interactions (DDIs), elderly person**	416 (100)
Without DDI	100 (24.0)
With 1–5 DDIs	230 (55.3)
With 6–10 DDIs	63 (15.1)
With > 10 DDIs	23 (5.5)
**Drug-drug interactions (DDIs)**	1360 (100)
Contraindicated DDI	3 (0.2)
Major DDI	672 (49.4)
Moderate DDI	621 (45.7)
Minor DDI	64 (4.7)

**Data are presented as** n (%).

**Abbreviations:** PP, polypharmacy; PIM, potentially inappropriate medication; DDI, drug-drug interaction.

Four participants (1%) were on 4 PIMs. With regard to DDI, 23 participants (5.5%) were exposed to > 10 potential DDIs. Overall, 373 participants (89.7%) were exposed to at least 1 criteria of PP, PIM and/or DDI. Of these 373, 50 (12%) were exposed to one criterion, 142 (34.1%) to two criteria, and 181 (43.5%) to all three criteria.

A total of 657 PIMs were identified among the study population, as presented in Table 4. The most common prescribed PIM were for neurological and cardiovascular systems. Antipsychotics and benzodiazepines were the most frequently prescribed PIMs.

**Table 4:** Potentially inappropriate medications of the population study according to the 2019 Beers Criteria.

PIMs Independent of Medical Condition		PIMs due to Drug-Disease or Drug-Syndrome Interactions	
	(n = 657 PIMs)		(n = 657 PIMs)
System/Therapeutic category/drugs	N (%)	System/Therapeutic category/ drugs	N (%)
**According to systems**		**According to systems**	
Central nervous system	210 (37.7)	Cardiovascular system	2 (0.4)
Cardiovascular system	60 (10.8)	Syncope	2 (0.4)
Endocrine	7 (1.3)	Central nervous system	245 (44.0)
Pain medications	5 (0.9)	Delirium	60 (10.8)
**According to therapeutic categories**		Dementia or cognitive impairment	185 (33.2)
Peripheral alpha-1 blockers	16 (2.9)	History of falls or fractures	121 (21.7)
Antiarrhythmic agents	44 (7.9)	Gastrointestinal system	3 (0.5)
Barbiturates	1 (0.2)	Parkinson disease	4 (0.7)
Benzodiazepines	58 (10.4)	**According to therapeutic categories**	
Antipsychotics	119 (21.4)	AChEIs	2 (0.4)
Antidepressants	23 (4.1)	Antipsychotics	213 (38.2)
Other CNS alpha-agonists	1 (0.2)	Benzodiazepines	94 (16.9)
Anticholinergics	8 (1.4)	Anticholinergics	13 (2.3)
Estrogen	6 (1.1)	Corticosteriods	4 (0.8)
Sulfonylureas, long acting	1 (0.2)	Antiepileptics	10 (1.8)
Non-COX-2-selective NSAIDs	5 (0.9)	Antidepressants	35 (6.3)
**Total**	282 (42.9)	Tramadol	1 (0.2)
		Non-COX-2-selective NSAIDs	3 (0.5)
		**Total**	375 (57.1)

**Data are presented as** n (%).

**Abbreviation:** PIMs, Potentially inappropriate medications; AChEIs, Acetylcholinesterase inhibitors; CNS, central nervous system; Non-COX-2-selective NSAIDs, Non-cyclooxygenase-2-selective non-steroidal anti-inflammatory drugs.

In the current study, 1360 medication combinations resulted in potential DDIs, as shown in Tables 3 and 5. The majority of the participants (49.4%) were exposed to major DDIs. Three single pairwise of drug combinations contributed to contraindicated DDIs, for example, the combination of haloperidol with metoclopramide, citalopram with metoclopramide and oxybutynin with potassium. The drug combination frequently implicated in potential DDI was that of heparin with aspirin, the interactive effect of which increases the risk of bleeding.

**Table 5:** Lists of contraindicated drug interactions and most common major drug interactions with potential effects according to Micromedex Drug Interaction Database.

Drug combination	Potential effects	(n = 1360) N (%)
**Contraindicated drug interactions**		
Haloperidol + Metoclopramide	Increased risk of extrapyramidal reaction and neuroleptic malignant syndrome	1 (0.1)
Citalopram + Metoclopramide	Increased risk of extrapyramidal reaction and neuroleptic malignant syndrome	1 (0.1)
Oxybutynin + Potassium	Increased risk of gastrointestinal lesions	1 (0.1)
**Major drug interactions**		
Heparin + Aspirin	Increased risk of bleeding	82 (6.0)
Aspirin + Furosemide	Reduced diuretic effectiveness and possible nephrotoxicity	52 (3.8)
Polyethylene glycol + Senna	Increased risk of mucosal ulceration or ischemic colitis	39 (2.9)
Aspirin + Metformin	Increased risk of hypoglycemia	20 (1.5)
Aspirin + Digoxin	Increased serum concentration of digoxin; prolonged half-life of digoxin	17 (1.3)
Clopidogrel + Heparin	Increased risk of bleeding	14 (1.0)
Sertraline + Aspirin	Increased risk of bleeding	12 (0.9)
Citalopram + Aspirin	Increased risk of bleeding	12 (0.9)
Citalopram + Heparin	Increased risk of bleeding	12 (0.9)
Clopidogrel + Omeprazole	Reduced plasma concentrations of clopidogrel active metabolite and reduced antiplatelet activity	12 (0.9)

**Data are presented as** n (%).

### Factors associated with the exposure to triad combination

After univariate logistic regression analyses were performed, significant variables for the exposure to all three criteria of PP, PIM and DDI were identified, as shown in Table 6. These significant variables, age, sex, number of comorbidities, short ADL at admission, premorbid IADL were added to adjust multivariate logistic model. After this adjustment, the independent risk factors of triad combination were the presence of diagnosis of atrial fibrillation (OR = 2.12 [1.93–3.96]) and increased medication use in cardiac therapy (OR = 3.49 [1.76–6.92]), psycholeptics (OR = 7.09 [4.23–11.87]) and psychoanaleptics (OR = 2.41 [1.44–4.03]). However, the degree of cognitive impairment, age, sex, ADLs and number of comorbidities were not associated factors for the exposure to triad combination of PP, PIM and DDI.

**Table 6:** Logistic regression model of factors associated with exposure to triad combination of PP, PIM and DDI.

Variables	Unadjusted OR (95%CI)	*P*- value	Adjusted OR^a^ (95%CI)	*P*- value
**Patient characteristics**				
Age	0.99 (0.97–1.02)	0.790		
Female	0.81 (0.55–1.19)	0.279		
Short ADL scale	1.03 (0.99–1.07)	0.170		
Premorbid IADLs	1.02 (1.00–1.04)	0.036		
Number of comorbidities	1.19 (1.07–1.31)	0.001		
Diabetes mellitus	1.88 (1.16–3.07)	0.011		
Coronary artery disease	1.56 (1.03–2.38)	0.037		
Atrial fibrillation	2.07 (1.27–3.39)	0.004	2.12 (1.93–3.96)	0.019
Osteoporosis	1.76 (1.04–3.01)	0.037		
**Medications characteristics**				
A02 Drugs for acid related disorders	1.65 (1.09–2.49)	0.018		
A10 Drug used in diabetes	2.03 (1.16–3.57)	0.014		
A12 Mineral supplements	1.74 (1.16–2.62)	0.008		
C01 Cardiac therapy	3.99 (2.30–6.95)	<0.001	3.49 (1.76–6.92)	<0.001
C03 Diuretics	2.43 (1.50–3.93)	<0.001		
C09 Agents acting on the renin- angiotensin system	1.68 (1.13–2.49)	0.011		
C10 Lipid modifying agents	1.71 (1.14–2.56)	0.010		
H03 Thyroid therapy	1.94 (1.07–3.54)	0.030		
N03 Antiepileptics	2.64 (1.19–5.83)	0.016		
N05 Psycholeptics	4.88 (3.15–7.55)	<0.001	7.09 (4.23–11.87)	<0.001
N06 Psychoanaleptics	2.63 (1.70–4.05)	<0.001	2.41 (1.44–4.03)	0.001

^a^ Adjusted for age, sex, number of comorbidities, short ADL scale, premorbid IADLs and significant variable from unadjusted model.

**Data are presented as** odds ratio (95% confidence interval).

**Abbreviations:** ADL, activities of daily living; IADL, instrumental ADL; OR, odds ratio; CI, confidence interval.

### Factors associated with polypharmacy

The results of the multivariate logistic regression analyses for independent risk factors of polypharmacy are presented in Table 7. The findings revealed that the presence of the diagnosis of gastroesophageal reflux disease (GERD) (OR = 6.03 [1.64–22.09]) and that of DDI (OR = 34.16 [9.59–121.69]) were associated with increased polypharmacy. Moreover, a greater number of prescribed medications for constipation, mineral supplements, antithrombotic agents, diuretics, agents acting on the renin-angiotensin system, lipid modifying agents, antibacterial drugs, analgesics and psycholeptics were strong associated risk factors of polypharmacy (P<0.001).

**Table 7:** Logistic regression model of factors associated with exposure to each criteria of PP, PIM and DDI.

	Polypharmacy	PIM	DDI
Variables	Adjusted OR (95%CI)^a^	Adjusted OR (95%CI)^a^	Adjusted OR (95%CI)^a^^S^
**Patient characteristics**			
Number of comorbidities			1.24 (1.03–1.48)*
History of GERD	6.03 (1.64–22.09)**		
**Medications characteristics**			
Number of medications	-^b^		1.46 (1.17–1.82)**
A06 Drugs for constipation	14.21 (4.16–48.60) **		
A11 Vitamins	3.73 (1.25–11.17)*		
A12 Mineral supplements	10.82 (2.29–51.09) **		
B01 Antithrombotic agents	5.15 (1.63–16.28) **		
C01 Cardiac therapy		3.47 (1.80–6.66)**	
C02 Antihypertensives		6.24 (1.26–30.83) *	
C03 Diuretics	120.83 (8.70–1678.36) **		12.72 (1.64–98.68)*
C08 Calcium channel blockers	7.48 (1.42–39.45) *		
C09 Agent acting on the renin- angiotensin system	9.81 (2.80–34.37) **		
C10 Lipid modifying agents	14.55 (3.29–64.16) **		
J01 Antibacterial drugs	29.29 (4.79–178.80) **		
N02 Analgesics	10.13 (2.97–34.52) **		
N05 Psycholeptics	19.71 (4.88–79.64) **	18.38 (9.73–34.73) **	
N06 Psychoanaleptics		2.26 (1.30–3.93) **	6.14 (2.35–16.08)**
Presence of PP	-^b^		3.04 (1.12–8.27)*
Presence of PIM	0.30 (0.09–1.01)	-^b^	2.07 (1.07–4.00)*
Presence of DDI	34.16 (9.59–121.69) **	1.97 (1.08–3.62)*	-^b^

^a^ Adjusted for age, sex, number of comorbidities, short ADL scale, premorbid IADLs and significant variable from unadjusted model

* *P*<0.05

**P<0.01

-^b^ Not include in adjusted analyses.

**Data are presented as** odds ratio (95% confidence interval).

**Abbreviations:** GERD, gastroesophageal reflux disease; PP, polypharmacy; PIM, potentially inappropriate medication; DDI, drug-drug interaction; OR, odds ratio; CI, confidence interval.

### Factors associated with inappropriate medication use

The adjusted logistic regression analyses presented a greater risk of PIM which substantially increased with the condition of higher medication use in cardiac therapy (OR = 3.47 [1.80–6.66]), antihypertensives (OR = 6.24 [1.26–30.83]), psycholeptics (OR = 18.38 [9.73–34.73]) and psychoanaleptics (OR = 2.26 [1.30–3.93]), as presented in Table 7. Furthermore, the occurrence of DDI was associated with PIM (OR = 1.97 [1.08–3.62]).

### Factors associated with drug-drug interactions

According to multivariate logistic regression analyses, associated independent risk factors of greater DDI were a higher number of comorbidities (OR = 1.24 [1.03–1.48]) and prescribed medications (OR = 1.46 [1.17–1.82]) as well as the presence of polypharmacy (OR = 3.04 [1.12–8.27]) and PIM (OR = 2.07 [1.07–4.00]). The prescriptions of diuretics (OR = 12.72 [1.64–98.68]) and psychoanaleptics (OR = 6.14 [2.35–16.08]) marginally increased the prevalence of DDI, as presented in Table 7.

## Discussion

The current study has reported the risk factors associated with the exposure to three criteria or triad combination of PP, PIM and DDI and has explored the prevalence and characteristics of PP, PIM and DDI among the older people living with dementia admitted to an acute care setting during the period from 2006 to 2016. The prevalence of dementia in hospitalized elderly patients aged 65 and over was 13.9%, similar to the results of previous studies [74,75]. With regard to primary diagnosis, consistent with the study of Toot et al and Andrieu et al, patients having behavioural and psychological symptoms of dementia (BPSD) are related to an increased risk of hospitalization [76,77] as a result of caregivers’ stress and burdens [78,79]. In addition, falls and urinary tract infection are also common reasons for admission of people living with dementia, which is in line with previous studies [17,79–88]. However, falls and urinary tract infection could have been prevented in this geriatric population. Amongst patients diagnosed with dementia, a higher use of indwelling catheters, dehydration, poor hygiene and urinary incontinence may be associated with an increased risk of urinary tract infection (UTI) [89–92]. Furthermore, in terms of neuropsychiatric treatment, sedatives or antipsychotics are widely prescribed, which may exacerbate urinary retention and reduce the patient’s activity, leading to worsening UTI. Falls are common issues in these patients owing to having impaired visuospatial and executive functions as well as developing negative health outcomes from some medications to maintain cognitive functions and behavioral symptoms [93]. The most common co-morbidities are hypertension, followed by infection and coronary artery disease. The results of current study are similar to common combinations of chronic diseases found in the previous studies [94–97]. The most common pre-existing comorbidities with dementia such as hypertension and cardiovascular diseases could be explained that they are the predictors of the risk of dementia [95,96,98]. Compared to the non-triad combination group, the number of patients having diabetes mellitus, coronary artery disease and atrial fibrillation in the triad combination were substantially higher. Due to management, most patients with these chronic diseases were treated with multiple medications and with narrow therapeutic medications such as digoxin and warfarin, leading to an increased risk of the exposure to three criteria of PP, PIM and DDI [98].

The most frequently prescribed medication categories were antithrombotic agents and agents acting on the renin-angiotensin system which were prescribed for treatment of common chronic diseases such as hypertension and coronary artery diseases [99]. Furthermore, antithrombotic agents were widely prescribed for patients with hip fractures to prevent venous thromboembolism [54]. Comparing with non-triad combination group, the number of medications use in cardiac therapy, diuretics, psycholeptics and psychoanaleptics in triad combination group were substantially higher.

As would be expected, hospitalized older patients with dementia are prone to be prescribed five or more medications daily [41,100], which are associated with higher co-morbidities and greater demented-related complications [33,40]. In accordance to the study of Brunet et al and Von-Ranteln Kruse et al, between 82–98% of hospitalized older people living with dementia experienced polypharmacy [55,56]. With regard to factors associated with polypharmacy, a higher number of prescribed medication in cardiac therapy, psycholeptics and psychoanaleptics were revealed. The findings from previous studies contribute to a growing body of evidence which demonstrates the presence of drug interactions associated with a higher risk of polypharmacy [101–103]. According to Park H-Y el al’s study [104] polypharmacy is associated with the increase of comorbidities, which was not seen in our study.

In the case of inappropriate medication use, more than half of this geriatric population were more likely to be exposed to at least 1 PIM. This prevalence was higher than the results of previous studies [57,105,106]. However, the variability in prevalence of PIM depends on the population characteristics, healthcare settings and the PIMs lists used such as the Beer Criteria, the STOPP/START criteria, PRISCUS list and Laroche list [71,107–109]. Regarding characteristics of PIMs according to the 2019 Beers criteria, the most common prescribed PIM classifications are cardiovascular system medications including cardiac therapy (antiarrhythmic agents) and central nervous system medications including psycholeptics and psychoanaleptics. These findings can explain why using of cardiac therapy, psycholeptics and psychoanaleptics are strong predictors of the occurrence of PIM. According to the 2019 Beers criteria, antipsychotics and benzodiazepines are considered common prescribed PIMs which may result in worsening of cognitive function and detrimental health consequences [71,110–116]. Furthermore, antipsychotics are known as an increased risk of cardiovascular events (stroke and heart arrhythmia) and mortality [117–120] whereas benzodiazepines are labeled as the predictor of falls and fractures [121]. However, these PIMs could have been prevented if medication prescribing is carefully balanced between risks and benefits and is avoided for unsuitable patients. Although some dispensed medications such as antipsychotics and benzodiazepines could relieve BPSD, these medications should not be used as the first line treatment [114]. Therefore, precipitating causes should be managed and non-pharmacological treatment should be attempted before the initiation of antipsychotics [122,123]. In contrast to previous studies [39,124–126], PP and number of medications were not linked to the use of PIMs in this study. In dementia, common risk factors of PIMs are the use of medications to relieve BPSD such as psycholeptics and psychoanaleptics. Unlike our results, other studies [39,127] have shown the associations of some medical conditions, such as urinary incontinence and osteoarthritis, with the use of PIMs.

The prevalence of DDI in this geriatric population with dementia in acute care setting was substantially higher than those prevalence in a previous study [60]. In this study, more than one third of elderly patients with dementia were exposed to major potential DDI, which is in line with Bogetti-Salazar ‘s study [128]. The most common potential DDI was the combination of heparin with aspirin which are prescribed to treat common comorbidities such as cardiovascular diseases. This common drug combination, in the study could be prescribed to prevent venous thromboembolism as complication of falls and fractures. This study demonstrates that the more prescribed medications patients take, the greater risks of DDIs they experience [60,101,128]. Furthermore, taking of PIMs and PP poses a significant risk of drug interactions [101,129]. Additionally, specific medication classifications including psychoanaleptics (antidepressants and anti-dementia drugs) and diuretic are associated with a higher risk of DDI due to age-related pharmacokinetic and pharmacodynamic changes [60,128,130,131]. However, no relationship was found between DDI and any specific medical condition factor, sex, age, activity of daily living [60,128].

In terms of exposure to triad combination, older patients diagnosed with dementia are prone to triad combination of PP, PIM and DDI. In the context of predictors of triad combination, older patients with dementia having atrial fibrillation were found to be more likely to be exposed to triad combination of PP, PIM and DDIs as compared to those without atrial fibrillation. In treatment of atrial fibrillation and prophylaxis complications, many types of medications such as antiarrhythmic drugs and antithrombotic agents are prescribed as a combination of drugs of choice. These medications have significant narrow therapeutic index and dose-response variability, which may contribute to increased risk of PIM and DDI [132]. Additionally, an increased use of cardiac therapy, psychoanaleptics and psycholeptics were important risk factors of the exposure to triad combination of PP, PIM and DDI.

Medication management should vary based on individuals and prescribers should carefully consider patients’ preferences for treatment as well as taking into account the risks and benefits of medication prescribing. Moreover, raising practitioners’ awareness regarding careful medication use is essential to avoid undesirable drug problems such as PIMs and DDIs, the great proportion of which could be prevented. Therefore, strategies of comprehensive medication reviews and medication reconciliation by physicians and pharmacists should be initiated to optimize medication prescribing for hospitalized elderly patients with dementia in order to limit the occurrence of PP, PIM and DDI.

### The strengths and limitations

To our knowledge, this is the first study to explore risk factors associated with the exposure to the triad combination of PP, PIM and DDI and to evaluate the prevalence and characteristics of PP, PIM and DDI among older patients with dementia in an acute care setting during a ten-year period. Furthermore, a standardized interRAI Acute Care (AC) assessment tool was used to gather clinical information, social attributes and medication prescribing profiles of the geriatric population with dementia. Trained nurse assessors gathered all comprehensive health information data and conducted many clinical assessments for dementia to refer to interRAI acute care (AC) databases, as standard assessment database for acute care setting. The participants were determined by using a combination of medical records with standard cognitive testing including CPS scores ranging from 2 to 4 in order to support accuracy in dementia diagnosis.

Several limitations of this study have to be taken into consideration. First, this study was conducted only in older patients with dementia referred for specialist geriatric consultation in a single acute care setting; therefore, the findings may not be applicable to older persons admitted to other facilities or who are not diagnosed with dementia. Patients are generally referred for geriatric assessment for complex care needs and multimorbidity as well as decisions around discharge to residential aged care facilities, admission to transition care programs, and potential for rehabilitation. Not all patients with dementia are referred for specialist consultation and so the number enrolled in this study is likely to be an underestimation of the number of older patients with dementia admitted to hospital. Second, the number of older patients with dementia may be underestimated due to under-diagnosis before admission [12,17,20,21]. Third, the identification of dementia subtypes may present differences in the results, but dementia was not classified following etiologies in this study. Fourth, the absence of detailed duration and indication of medication prescribing during admission may result in underestimation of PIMs. Fifth, clinical relevant outcomes due to drug interactions were not taken into account. Furthermore, all drug interactions were considered only by single pairwise of drug combination, but interactions from three drug combination or more were not accounted for. Finally, this study did not address other common DRPs among the elderly with dementia, such as ADRs [44].

## Conclusion

The exposure to triad combination of PP, PIM and DDI are common among people with dementia as a result of their vulnerable conditions and the greater risks of adverse events from medications use. This study identified the use of cardiac therapy, psycholeptics and psychoanaleptics as predictors of exposure to PP, PIM and DDI. Therefore, use of these medications should be carefully considered and closely monitored. Furthermore, comprehensive medication reviews to optimize medication prescribing should be initiated and continually implemented for this vulnerable population.

## Supporting information

S1 File Potentially inappropriate medications among older adults living with dementia. (DOC)

S2 File Drug interactions among older adults living with dementia. (DOC)
