# Supplementary material for: Drug related problems in older adults living with dementia
Source: PLoS One. 2020 Jul 31;15(7):e0236830. doi: 10.1371/journal.pone.0236830 (PMC7394402; doi:10.1371/journal.pone.0236830)
Supplement: S1 File — (DOC) [file pone.0236830.s001.doc]

**S1. Potentially inappropriate medications among older adults living with dementia**

| **PIMs Independent of Medical Condition** | |
| --- | --- |
| **Organ system/ Therapeutic Category/Drugs** | **Number (N)** |
| **Cardiovascular** | **60** |
| Antiarrhythmic drugs | 44 |
| Peripheral alpha-1 blockers | 16 |
| **Central nervous system** | **210** |
| Barbiturate | 1 |
| Benzodiazepines | 58 |
| Antipsychotics | 119 |
| Antidepressants | 23 |
| SSRIs | 7 |
| TCAs | 16 |
| Other CNS alpha-agonists | 1 |
| Anticholinergics | 8 |
| Antimuscarinics | 6 |
| Antiemetics | 1 |
| Antiparkinsonian agents | 1 |
| **Endocrine** | **7** |
| Estrogen | 6 |
| Sulfonylureas, long acting | 1 |
| **Pain medications** | **5** |
| Non-cyclooxygenase-2-selective NSAIDs, oral | 5 |
| **Total** | **282** |

**Abbreviation:** PIMs, Potentially inappropriate medications; SSRIs, selective serotonin reuptake inhibitors; TCAs; tricyclic antidepressants; CNS, central nervous system; Non-COX-2-selective NSAIDs, Non-cyclooxygenase-2-selective non-steroidal anti-inflammatory drugs

**S1. Potentially inappropriate medications among older adults** living with dementia

| **PIMs due to Drug-disease or Drug-syndrome interactions** | |
| --- | --- |
| **Organ system/ Therapeutic Category/Drugs** | **Number (N)** |
| **Cardiovascular system** | **2** |
| **Syncope** | **2** |
| AChEIs | 2 |
| **Central nervous system** | **245** |
| **Delirium** | **60** |
| Benzodiazepines | 14 |
| Antipsychotics | 37 |
| Anticholinergics | 5 |
| Corticosteroids | 4 |
| **Dementia or cognitive impairment** | **185** |
| Antipsychotics | 119 |
| Anticholinergics | 8 |
| Benzodiazepines | 58 |
| **History of fall or fractures** | **121** |
| Antiepileptics | 10 |
| Antipsychotics | 53 |
| Benzodiazepines | 22 |
| Antidepressants | 35 |
| TCAs | 11 |
| SSRIs | 21 |
| SNRIs | 3 |
| Tramadol | 1 |
| **Parkinson disease** | **4** |
| Antipsychotics | 4 |
| **Gastrointestinal** | **3** |
| History of gastric or duodenal ulcers | 3 |
| Non-COX-2-selective NSAIDs | 3 |
| **Total** | **375** |

**Abbreviation:** PIMs, Potentially inappropriate medications; AChEIs, Acetylcholinesterase inhibitors; SSRIs, selective serotonin reuptake inhibitors; SNRIs, serotonin norepinephrine reuptake inhibitors; TCAs; tricyclic antidepressants; CNS, central nervous system; Non-COX-2-selective NSAIDs, Non-cyclooxygenase-2-selective non-steroidal anti-inflammatory drugs
