# Supplementary material for: Drug related problems in older adults living with dementia
Source: PLoS One. 2020 Jul 31;15(7):e0236830. doi: 10.1371/journal.pone.0236830 (PMC7394402; doi:10.1371/journal.pone.0236830)
Supplement: S2 File — (DOC) [file pone.0236830.s002.doc]

**S2. Drug drug interactions among older adults living with dementia**

| **Drug drug interactions** | **Number (N)** |
| --- | --- |
| Acetaminophen+phenytoin | 2 |
| Alendronate+calcium | 17 |
| Albuterol+furosemide | 2 |
| Alendronate+ferrous | 3 |
| Amiodarone+digoxin | 1 |
| Amiodarone+irbesartan | 1 |
| Amiodarone+venlafaxine | 1 |
| Amiodarone+haloperidol | 1 |
| Amiodarone+risperidol | 2 |
| Amiodarone+simvastatin | 2 |
| Amiodarone+atorvastatin | 2 |
| Amitriptyline+oxycodone | 2 |
| Amitriptyline+risperidol | 3 |
| Amlodipine+clopidogrel | 4 |
| Amlodipine+digoxin | 3 |
| Amlodipine+simvastatin | 4 |
| Amoxicillin+venlafaxine | 1 |
| Amoxicillin+gentamicin | 1 |
| Aspirin+furosemide | 52 |
| Aripiprazole+oxycodone | 1 |
| Aspirin+aluminium hydroxide | 1 |
| Aspirin+amitriptyline | 6 |
| Aspirin+calcium | 35 |
| Aspirin+digoxin | 17 |
| Aspirin+dothiepin | 1 |
| Aspirin+fluoxetine | 1 |
| Aspirin+meloxicam | 1 |
| Aspirin+metformin | 20 |
| Aspirin+perindopril | 27 |
| Aspirin+valproate | 5 |
| Aspirin+pindolol | 1 |
| Aspirin+sodium bicarbonate | 2 |
| Aspirn+venlafaxine | 5 |
| Aspirn+spironolactone | 8 |
| Aspirin+propanolol | 1 |
| Atenolol+aspirin | 17 |
| Atenolol+metformin | 2 |
| Atenolol+digoxin | 3 |
| Atenolol+calcium | 6 |
| Atorvastatin+digoxin | 5 |
| Atorvastatin+cyclosporin | 1 |
| Allopurinol+cyclosporin | 1 |
| Ascorbic+cyanocobalamin | 2 |

**S2. Drug drug interactions among older adults living with dementia**

| **Drug drug interactions** | **Number (N)** |
| --- | --- |
| Bisoprolol+aspirin | 15 |
| Bisoprolol+digoxin | 8 |
| Bisoprolol+insulin | 3 |
| Bisacodyl+polyethylene glycol | 1 |
| Bromazepam+buprenorphrine | 1 |
| Bromazepam+haloperidol | 1 |
| Bromazepam+temazepam | 1 |
| Buprenorphine+nitrazepam | 1 |
| Bisacodyl+calcium | 1 |
| Buprenorphine+risperidol | 1 |
| Buprenorphine+paroxetine | 1 |
| Buprenorphine+amitriptyline | 1 |
| Buprenorphine+clopidogrel | 1 |
| Buprenorphine+venlafaxine | 1 |
| Calcium+digoxin | 10 |
| Calcium+indapamide | 1 |
| Calcium+strontium | 6 |
| Calcium+verapamil | 2 |
| Carvedilol+metformin | 2 |
| Carvedilol+aspirin | 3 |
| Carvedilol+valproate | 1 |
| Citalopram+omeprazole | 10 |
| Citalopram+aspirin | 12 |
| Citalopram+donepezil | 1 |
| Citalopram+heparin | 12 |
| Citalopram+dipyridamole | 1 |
| Citalopram+metoclopramide | 1 |
| Citalopram+risperidol | 6 |
| Clopidogrel+aspirin | 9 |
| Clopidogrel+escitalopram | 3 |
| Clopidogrel+atorvastatin | 11 |
| Clopidogrel+heparin | 14 |
| Clopidogrel+omeprazole | 12 |
| Clopidogrel+codein | 1 |
| Clopidogel+venlafaxine | 1 |
| Cyclosporin+aspirin | 1 |
| Ciprofloxacin+glicazide | 1 |
| Ciprofloxacin+insulin | 1 |
| Ciprofloxacin+warfarin | 1 |
| Ciprofloxacin+duloxetine | 1 |
| Carbamazepine+haloperidol | 1 |
| Carbamazepine+furosemide | 2 |

**S2. Drug drug interactions among older adults living with dementia**

| **Drug drug interactions** | **Number (N)** |
| --- | --- |
| Carbamazepine+acetaminophen | 4 |
| Carbamazepine+risperidol | 1 |
| Carbamazepine+amitriptyline | 2 |
| Carbamazepine+ergocalciferol | 1 |
| Cyanocobalamin+omeprazole | 5 |
| Ciprofloxacin+prednisolone | 1 |
| Cholecalciferol+aluminum | 1 |
| Cyclosporin+mycophenolate | 1 |
| Cholestyramine+acetaminophen | 1 |
| Ciprofloxacin+calcium | 1 |
| Ciprofloxacin+metformin | 1 |
| Clopidogrel+felodipine | 2 |
| Desvenlafaxine+enoxaparin | 1 |
| Digoxin+furosemide | 21 |
| Digoxin+flecainide | 2 |
| Digoxin+metoclopramide | 1 |
| Digoxin+spironolactone | 6 |
| Digoxin+omeprazole | 12 |
| Digoxin+senna | 9 |
| Digoxin+metformin | 2 |
| Digoxin+albuterol | 1 |
| Dipyridamole+heparin | 6 |
| Dipyridamole+venlafaxine | 2 |
| Domperidone+ondansetron | 1 |
| Donepezil+duloxetine | 1 |
| Donepezil+fentanyl | 2 |
| Donepezil+olanzapine | 2 |
| Donepezil+oxybutynin | 2 |
| Donepezil+sertraline | 2 |
| Donepezil+tiotropium | 1 |
| Donepezil+venlafaxine | 1 |
| Donepezil+risperidol | 1 |
| Donepezil+amiodarone | 1 |
| Donepezil+amitriptyline | 1 |
| Donepezil+citalopram | 3 |
| Donepezil+desvenlafaxine | 1 |
| Donepezil+doxepin | 1 |
| Doxepin+aspirin | 2 |
| Doxepin+codein | 1 |
| Doxepin+trimethoprim | 1 |
| Doxepin+albuterol | 1 |
| Doxepin+risperidol | 1 |
| Diazepam+omeprazole | 4 |

**S2. Drug drug interactions among older adults living with dementia**

| **Drug drug interactions** | **Number (N)** |
| --- | --- |
| Diazepam+amitriptyline | 1 |
| Diazepam+oxycodone | 1 |
| Duloxetine+heparin | 1 |
| Duloxetine+aspirin | 1 |
| Desatinib+escitalopram | 1 |
| Enalapril+trimethoprim | 1 |
| Enalapril+aspirin | 1 |
| Enoxaparin+clopidogrel | 5 |
| Enoxaparin+sertraline | 1 |
| Enoxaparin+rivaroxaban | 1 |
| Enoxaparin+dipyridamole | 2 |
| Enoxaparin+escitalopram | 5 |
| Enoxaparin+piroxicam | 1 |
| Enoxaparin+venlafaxine | 1 |
| Escitalopram+oxycodone | 1 |
| Escitalopram+esomeprazole | 3 |
| Esomeprazole+fluconazole | 1 |
| Esomeprazole+levothyroxine | 1 |
| Estrogen(Estradiol)+phenytoin | 1 |
| Escitalopram+heparin | 3 |
| Escitalopram+aspirin | 2 |
| Felodipine+digoxin | 1 |
| Fentanyl+amitriptyline | 1 |
| Fentanyl+oxycodone | 1 |
| Fentanyl+midazolam | 1 |
| Ferrous+omeprazole | 11 |
| Ferrous+levofloxacin | 1 |
| Ferrous+levothyroxine | 4 |
| Ferrous+calcium | 10 |
| Ferrous+pantoprazole | 4 |
| Fosinopril+trimethoprim | 2 |
| Fosinopril+insulin | 2 |
| Fosinopril+spironolactone | 1 |
| Fosinopril+aspirin | 4 |
| Fosinopril+furosemide | 2 |
| Fosimopril+potassium | 1 |
| Furosemide+oxycodone | 1 |
| Furosemide+cholestyramine | 1 |
| Fluoxetine+risperidol | 1 |
| Flecainide+amitriptyline | 1 |
| Glicazide+metoprolol | 4 |
| Glicazide+furosemide | 5 |
| Glicazide+insulin | 2 |

**S2. Drug drug interactions among older adults living with dementia**

| **Drug drug interactions** | **Number (N)** |
| --- | --- |
| Glicazide+perindopril | 1 |
| Glicazide+aspirin | 6 |
| Glemiperide+aspirin | 1 |
| Galantamine+quetiapine | 1 |
| Galantamine+dothiepin | 1 |
| Galantamine+escitalopram | 1 |
| Haloperidol+amitriptyline | 2 |
| Haloperidol+escitalopram | 2 |
| Haloperidol+citalopram | 1 |
| Haloperidol+metoclopramide | 1 |
| Haloperidol+benztropine | 1 |
| Haloperidol+propanolol | 1 |
| Haloperidol+norfloxacin | 1 |
| Haloperidol+risperidol | 11 |
| Haloperidol+fluvoxamine | 1 |
| Haloperidol+roxithromycin | 1 |
| Haloperidol+trimethoprim | 2 |
| Heparin+aspirin | 82 |
| Heparin+venlafaxine | 3 |
| Heparin+ibuprofen | 3 |
| Hydrochlorothiazide+propanolol | 1 |
| Hydralazine+furosemide | 2 |
| Indapamide+aspirin | 2 |
| Indapamide+albuterol | 1 |
| Insulin+furosemide | 15 |
| Insulin+aspirin | 8 |
| Insulin+lisinopril | 1 |
| Insulin+atenolol | 2 |
| Insulin+carvedilol | 1 |
| Insulin+irbesartan | 1 |
| Insulin+metformin | 6 |
| Insulin+metoclopramide | 3 |
| Ibuprofen+lisinopril | 1 |
| Ibuprofen+aspirin | 1 |
| Ibuprofen+atenolol | 1 |
| Irbesartan+spironolactone | 1 |
| Levothyroxine+calcium | 9 |
| Levothyroxine+omeprazole | 13 |
| Lisinopril+aspirin | 3 |
| Lisinopril+metformin | 1 |
| Lisinopril+furosemide | 1 |
| Lithium+lactulose | 1 |
| Lithium+aspirin | 3 |

**S2. Drug drug interactions among older adults living with dementia**

| **Drug drug interactions** | **Number (N)** |
| --- | --- |
| Lithium+venlafaxine | 2 |
| Lithium+risperidol | 1 |
| Lithium+buprenorphine | 1 |
| Lamotrigine+escitalopram | 1 |
| Lamotrigine+acataminophen | 1 |
| Methimazole+atenolol | 2 |
| Methotrexate+sulfa | 1 |
| Methotrexate+aspirn | 1 |
| Methotrexate+lefunamide | 1 |
| Methotrexate+omeprazole | 3 |
| Methotrexate+ciprofloxacin | 1 |
| Methotrexate+foilc | 5 |
| Methotrexate+amoxicillin | 1 |
| Metoprolol+aspirin | 38 |
| Metoprolol+venlafaxine | 1 |
| Metoprolol+prazosin | 1 |
| Metoprolol+digoxin | 9 |
| Metoprolol+insulin | 6 |
| Metoprolol+citalopram | 3 |
| Metoprolol+fluoxetine | 1 |
| Metoprolol+rifampin | 1 |
| Metoprolol+metformin | 6 |
| Metoprolol+prazosin | 2 |
| Morphine+haloperidol | 1 |
| Methimazole+atenolol | 1 |
| Midazolam+oxycodone | 1 |
| Methyldopa+ferrous | 1 |
| Meloxicam+heparin | 1 |
| Mirtazapine+temazepam | 1 |
| Mirtazapine+fentanyl | 1 |
| Metformin+bisoprolol | 2 |
| Nitrofurantoin+foilc | 1 |
| Nitrazepam+buprenorphine | 1 |
| Olanzapine+haloperidol | 1 |
| Olanzapine+quetiapine | 1 |
| Olanazapine+nitrazepam | 1 |
| Olanzapine+buprenorphine | 1 |
| Oxazepam+oxycodone | 3 |
| Oxybutynin+potassium | 1 |
| Oxycodone+mirtazapine | 1 |
| Oxycodone+temazepam | 3 |
| Oxycodone+spironolactone | 1 |
| Oxycodone+haloperidol | 2 |

**S2. Drug drug interactions among older adults living with dementia**

| **Drug drug interactions** | **Number (N)** |
| --- | --- |
| Oxycodone+furosemide | 6 |
| Oxycodone+amitriptyline | 1 |
| Oxycodone+indapamide | 1 |
| Oxycodone+metoclopramide | 3 |
| Oxazepam+midazolam | 1 |
| Oxycodone+carbamazepine | 2 |
| Oxycodone+clopidogrel | 1 |
| Omega-3+heparin | 2 |
| Omega-3+clopidogrel | 1 |
| Omega-3+aspirin | 3 |
| Pantoprazole+levothyroxine | 3 |
| Pantoprazole+propanolol | 1 |
| Pantoprazole+warfarin | 1 |
| Paroxetine+fentanyl | 1 |
| Paroxetine+risperidol | 2 |
| Paroxetine+aspirin | 3 |
| Paroxetin+clopidogrel | 2 |
| Paroxitine+haloperidol | 2 |
| Paroxetine+heaprin | 6 |
| Paroxetine+dipyridamole | 1 |
| Paroxetine+amitriptyline | 1 |
| Perindopril+furosemide | 18 |
| Perindopril+insulin | 3 |
| Perindopril+heparin | 19 |
| Perindopril+indapamide | 1 |
| Perinopril+spironolactone | 2 |
| Phenobarbital+prednisolone | 1 |
| Phenytoin+valproic acid | 3 |
| Phenytoin+folic | 4 |
| Phenytoin+risperidol | 1 |
| Phenytoin+omeprazole | 2 |
| Phenytoin+aspirin | 1 |
| Pioglitazone+metformin | 1 |
| Piroxicam+prednisolone | 1 |
| Clopidogrel+citalopram | 1 |
| Polyethylene glycol+senna | 39 |
| Perindopril+potassium | 3 |
| Perindopril+metformin | 5 |
| Prednisolone+aspirin | 9 |
| Prednisolone+cyclosporin | 1 |
| Propanolol+ampicillin | 1 |
| Propanolol+calcium | 1 |
| Potassium+candesartan | 1 |

**S2. Drug drug interactions among older adults living with dementia**

| **Drug drug interactions** | **Number (N)** |
| --- | --- |
| Potassium+irbesartan | 1 |
| Prazosin+atenolol | 2 |
| Prazosin+carvediol | 1 |
| Prochloperazine+risperidol | 1 |
| Prochloperazine+citalopram | 1 |
| Phenytoin+atorvastatin | 1 |
| Phenytoin+risperidol | 1 |
| Phenytoin+estradiol | 1 |
| Phenytoin+nifedipine | 1 |
| Phenytoin+trimethoprim | 1 |
| Phenobarbital+verapamil | 1 |
| Phenobarbital+risperidol | 1 |
| Quetiapine+dothiepin | 1 |
| Quinopril+furosemide | 1 |
| Quetiapine+oxycodone | 3 |
| Quinapril+aspirin | 1 |
| Ramipril+metformin | 7 |
| Ramipril+glicazide | 2 |
| Ramipril+levofloxacin | 1 |
| Ramipril+lithium | 1 |
| Ramipril+candesartan | 1 |
| Ramipril+spironolactone | 3 |
| Ramipril+trimethoprim | 1 |
| Ramipril+furosemide | 13 |
| Rifampin+omeprazole | 1 |
| Ramipril+insulin | 5 |
| Ramipril+aspirin | 15 |
| Ramipril+potassium | 2 |
| Ranitidine+diltiazem | 1 |
| Ranitidine+aspirin | 2 |
| Risedronate+omeprazole | 5 |
| Rivastigmine+atenolol | 1 |
| Rifampicin+isoniazid | 1 |
| Rivaroxaban+verapamil | 1 |
| Rivaroxaban+aspirin | 1 |
| Sertraline+aspirin | 12 |
| Sertraline+heparin | 7 |
| Sertraline+amitriptyline | 1 |
| Sertraline+morphine | 3 |
| Sertraline+meloxicam | 1 |
| Sertraline+risperidol | 3 |
| Sertraline+haloperidol | 2 |
| Sertraline+ibuprofen | 1 |

**S2. Drug drug interactions among older adults living with dementia**

| **Drug drug interactions** | **Number (N)** |
| --- | --- |
| Sertraline+dipyridamole | 1 |
| Simvastatin+verapamil | 1 |
| Simvastatin+digoxin | 3 |
| Simvastatin+levothyroxine | 3 |
| Simvastatin+risperidol | 8 |
| Simvastatin+clopidogrel | 7 |
| Simvastatin+phenytoin | 2 |
| Simvastatin+carbamazepine | 1 |
| Simvastatin+colchicine | 1 |
| Simvastatin+diltiazem | 1 |
| Sitagliptin+metoprolol | 1 |
| Sitagliptin+furoemide | 2 |
| Sitagliptin+insulin | 1 |
| Sitagliptin+ramipril | 2 |
| Sitagliptin+glicazide | 1 |
| Sitagliptin+digoxin | 1 |
| Sitagliptin+metformin | 3 |
| Sulfa+azathioprine | 1 |
| Sulfa+risperidol | 1 |
| Sulfamethoxazone+insulin | 1 |
| Spironolactone+potassium | 1 |
| Sotalol+digoxin | 1 |
| Telmisartan+spironolactone | 1 |
| Trimethoprim+risperidol | 3 |
| Trimethoprim+telmisartan | 5 |
| Trimethoprim+amitriptyline | 1 |
| Trimethoprim+azathiprine | 1 |
| Trimethoprim+digoxin | 3 |
| Temazepam+morphine | 1 |
| Temazepam+risperidol | 1 |
| Temazepam+metoclopramide | 2 |
| Temazepam+codeine | 1 |
| Trandolapril+furosemide | 2 |
| Trandolapril+potassium | 2 |
| Trandolapril+aspirin | 2 |
| Tiotropium+doxepin | 1 |
| Tamsulosin+atenolol | 1 |
| Valproate+nifedipine | 4 |
| Valproate+risperidol | 4 |
| Vidagliptin+metformin | 1 |
| Vidagliptin+glicazide | 1 |
| Verapamil+metformin | 1 |
| Verapamil+atorvastatin | 3 |

**S2. Drug drug interactions among older adults living with dementia**

| **Drug drug interactions** | **Number (N)** |
| --- | --- |
| Verapamil+phenytoin | 2 |
| Warfarin+allopurinol | 2 |
| Warfarin+amoxicillin | 2 |
| Warfarin+levofloxacin | 1 |
| Warfarin+spironolactone | 2 |
| Warfarin+acetaminophen | 4 |
| Warfarin+amitriptyline | 1 |
| Warfarin+prednisolone | 1 |
| Warfarin+levothyroxine | 1 |
| Warfain+omeprazole | 5 |
| Warfarin+aspirin | 2 |
| Warfarin+amiodarone | 2 |
| Warfarin+rosuvastatin | 1 |
| Warfarin+cephalexin | 1 |
|  |  |
